# Trends of Surgical Service Utilization for Lumbar Spinal Stenosis in South Korea: A 10-Year (2010–2019) Cross-Sectional Analysis of the Health Insurance Review and Assessment Service—National Patient Sample Data

**DOI:** 10.3390/medicina59091582

**Published:** 2023-08-31

**Authors:** HyungWook Ji, Seungwon Shin, Yongjoo Kim, In-Hyuk Ha, Doori Kim, Yoon Jae Lee

**Affiliations:** 1 Jaseng Hospital of Korean Medicine, 536 Gangnam-daero, Gangnam-gu, Seoul 06110, Republic of Korea; hyungwook0510@naver.com; 2 College of Korean Medicine, Sangji University, Wonju 26339, Republic of Korea; ssw.kmd@gmail.com (S.S.); yongjookim@sangji.ac.kr (Y.K.); 3 Jaseng Spine and Joint Research Institute, Jaseng Medical Foundation, 540 Gangnam-daero, Hangnam-gu, Seoul 06110, Republic of Korea; hanihata@gmail.com

**Keywords:** lumbar spinal stenosis, HIRA claims data, national patient sample, decompression, fusion, surgical service utilization

## Abstract

*Background and Objectives*: This retrospective, cross-sectional, and descriptive study used claims data from the Korean Health Insurance Review and Assessment Service (HIRA) between 2010 and 2019 to analyze the trend of surgical service utilization in patients with lumbar spinal stenosis (LSS). *Materials and Methods*: The national patient sample data provided by the HIRA, which consisted of a 2% sample of the entire Korean population, was used to assess all patients who underwent decompression or fusion surgery at least once in Korea, with LSS as the main diagnosis from January 2010 to December 2019. An in-depth analysis was conducted to examine the utilization of surgical services, taking into account various demographic characteristics of patients, the frequency of claims for different types of surgeries, reoperation rates, the specific types of inpatient care associated with each surgery type, prescribed medications, and the overall expense of healthcare services. *Results*: A total of 6194 claims and 6074 patients were analyzed. The number of HIRA claims for patients increased from 393 (2010) to 417 (2019) for decompression, and from 230 (2010) to 244 (2019) for fusion. As for the medical expenses of surgery, there was an increase from United States dollar (USD) 867,549.31 (2010) to USD 1,153,078.94 (2019) for decompression and from USD 1,330,440.37 (2010) to USD 1,780,026.48 (2019) for fusion. Decompression accounted for the highest proportion (65.8%) of the first surgeries, but more patients underwent fusion (50.6%) than decompression (49.4%) in the second surgery. Across all sex and age groups, patients who underwent fusion procedures experienced longer hospital stays and incurred higher medical expenses for their inpatient care. *Conclusion*: The surgical service utilization of patients with LSS and the prescribing rate of opioids and non-opioid analgesics for surgical patients increased in 2019 compared to 2010. From mid-2010 onward, claims for fusion showed a gradual decrease, whereas those for decompression showed a continuously increasing trend. The findings of this study are expected to provide basic research data for clinicians, researchers, and policymakers.

## 1. Introduction

Lumbar spinal stenosis (LSS) is a representative condition involving degenerative spine changes caused by direct compression or inflammation of nerves or vascular structures inside the spinal canal for various reasons, resulting in spinal canal narrowing [1]. The typical symptoms of LSS include lower back pain, pain in the buttocks or legs when walking, and pain that worsens when leaning or bending backward [2,3]. A systemic review reported that the prevalence of LSS would be approximately 11% in the general population [4], and similar prevalence levels were also reported in Europe and the United States (U.S.) [4,5] While the prevalence of LSS in Korea has not been accurately estimated [6], a population-based cohort study in Japan reported that symptomatic LSS was present in approximately 10% of people with an increasing trend depending on age [7].

Treatment options for individuals with LSS can be broadly classified into surgical and non-surgical approaches. Non-surgical treatments encompass a range of modalities, including physical therapy, pharmacotherapy, injections, exercise therapy, and manual therapy techniques [8]. It is worth noting that LSS stands as the leading cause of spinal surgery in patients aged 65 years and above [9]. Surgical intervention becomes necessary for patients with spinal stenosis who have not responded to conservative treatment for two to three months and exhibit a rapid progression of neurological abnormalities or cauda equina syndrome [10]. The main types of surgeries for LSS are decompression and fusion. The primary objective of decompression is to alleviate pressure on the narrowed spinal canal and relieve compression on the nerve roots, thereby facilitating their release. Candidates for fusion surgery include those with multi-segmental stenosis over a wide area and those with radiological findings of degenerative spondylolisthesis, for which fusion is considered to achieve spinal stability [11]. 

The surgery rate of patients with LSS has shown an increasing trend. Notably, when considering the specific surgical procedures, the rate of fusion surgeries demonstrated a more pronounced and rapid increase compared to decompression procedures. A study conducted in Denmark from 2002 to 2018 examined surgery rates for LSS, revealing an overall annual surgery rate of 33% among diagnosed patients. During the study period, the rate of decompression without fusion increased by 128%, while the rate of decompression with fusion showed a larger increase of 199% [12]. Similarly, in the U.S., a study reported a 113% increase in lumbar fusion surgeries in 2001 compared to 1996 [13]. A study comparing surgery volumes for LSS between 2003 and 2008 in Korea indicated a remarkable 254% increase. Moreover, the rate of fusion procedures, as the chosen surgical method, rose from 20.3% in 2003 to 27% in 2008 [14]. Another study analyzing treatment costs in Korea found that between 2010 and 2019, the cost of surgical decompression increased by 1.6 times, while the cost of fusion surged approximately four times during the same period [15]. However, studies have reported that fusion is associated with higher surgical expenses, post-discharge healthcare costs, and an increased risk of major complications compared to decompression [9,16]. Moreover, a previous study reported that fusion was unlikely to result in superior clinical outcomes compared with decompression in patients with LSS [17].

As shown in previous studies, with the increasing prevalence of LSS and the growing number of patients choosing surgical approaches for treatment, the direct and indirect expense burden for those treatments has increased accordingly, warranting an in-depth analysis of recent trends in surgical treatment for patients with LSS. A five-year retrospective cohort study conducted in the U.S. reported that the rate of fusion procedures increased 15-fold, and that compared with decompression alone, fusion was associated with an increased risk of life-threatening complications and rehospitalization [17]. In addition, a five-year cohort study in Korea reported an increase in the surgical treatment rate for LSS by 2.54 times in 2008 compared to the rate in 2003 and an increase in the re-operation rate from 8.1% to 11.2% for the same period [14]. However, analyses of more recent, up-to-date trends are required compared to these previous studies, and a more detailed analysis of healthcare utilization is required for patients with LSS undergoing surgical treatment. 

Therefore, in this study, 10-year (2010–2019) data of nationwide health insurance claims provided by the Health Insurance and Review Assessment (HIRA) in Korea were used to comprehensively evaluate the status and trends of surgical treatment in patients with LSS by comparing different surgical methods of decompression and fusion. It is expected for this study to be effectively deployed by practitioners and policymakers.

## 2. Materials and Methods

### 2.1. Data Source

This study used 10-year (January 2010–December 2019) claims data from the national patient sample data of HIRA (HIRA-NPS). In Korea, the National Health Insurance Service (NHIS), a mandatory social health insurance system, provides health insurance coverage to approximately 97% of the national population. All healthcare providers (hospitals and clinics) in Korea submit their medical records, including diagnosis and procedure codes, to HIRA to receive reimbursement for their healthcare services. The HIRA-NPS data consists of a 2% sample (approximately 1,000,000 people) of the entire Korean population each year. A sex- and age-stratified random sampling method was used for this sampling. The utilization of claims data offers a significant advantage in healthcare and public health research because it provides representative information about the national population. This valuable dataset encompasses a wide range of parameters essential for research, including healthcare service status, diagnostic information, treatment records, surgical history, and medication prescriptions.

To ensure privacy and confidentiality, the raw data undergo a rigorous de-identification process, eliminating personal or identifiable information. This de-identification allows for effective sampling while safeguarding the privacy of individuals and entities involved. As a result, claims data can be effectively utilized in various approaches to advance healthcare and public health research. Stratified random samples of one-year segments underwent statistical processing to provide secondary data for this study. 

### 2.2. Study Design and Study Population

This was a retrospective, cross-sectional, descriptive study based on the 10-year HIRA-NPS data (January 2010 to December 2019). To extract information related to patients with LSS from the HIRA-NPS data, the disease codes were selected based on the seventh revision of the Korean standard classification of diseases (KCD-7). The KCD-7 is the Korean version of mapping of the International Classification of Disease and Causes of Death-10 (ICD-10). The diagnostic code for LSS was defined as M480 (spinal stenosis), and all subtypes of the code were included in this study.

Among patients with LSS as the main diagnosis, those undergoing spinal decompression or fusion surgery were selected as the study sample. For analysis of re-operation status, patients who underwent two or more spinal surgeries in the applicable year were chosen additionally.

The procedure codes for decompression or fusion surgeries included arthrodesis of the spine (N0466, N1466, N0469, N1469, N2470, and N1460), laminectomy (N1499 and N2499), diskectomy (N1493 and N1494), injection for chemonucleolysis (N1495), and aspiration of the nucleus pulposus of the intervertebral disk (N1496). 

If demographic information or healthcare providers’ information about the patients was missing, the data were excluded from the analysis in this study, and if the total treatment cost and days of treatment were recorded as zero or not entered, such data were also excluded from the study. 

The study protocol was approved by the HIRA Deliberative Committee for public data provision, and the study was conducted in accordance with relevant guidelines and regulations. The current study was reviewed and qualified for an exemption by the Institutional Review Board of Jaseng Hospital of Korean Medicine, Seoul, Republic of Korea (JASENG 2023-07-016).

### 2.3. Study Outcomes

The basic demographic characteristics of the patients were categorized into age, sex, payer type, type of visit, and type of medical institution. The age category was divided into five groups in 10-year increments for adults aged from <45 years to ≥75 years. The payer types encompassed NHIS, Medicaid, and other forms of coverage. Visits were categorized as either outpatient or inpatient visits. The types of medical institutions were classified into three main categories: tertiary hospitals, general hospitals, and hospitals or clinics. The categorization of age, sex, payer type, and type of medical institution was based on the number of claims and information in the HIRA claims. All these categories in the basic demographic characteristics were used to analyze patients with LSS using surgical services. For each year in the 10 years from 2010 to 2019, the total number of claims, the total expense of healthcare services, and the number of claims and expenses of healthcare services were analyzed by the category of demographic characteristics. 

The total expense of healthcare services included all medical expenses during the patient’s hospital stay concerning the claims of the surgical service received, and the details of the treatment and care received. The categories used for the expenses were as follows: examination, injection, medication and prescription, procedure/surgery, admission fee, anesthetics, radiology, examination, physical therapy, psychotherapy, uncovered items, and others. In addition, a more detailed analysis of the subtypes was performed by analyzing the rates of injection of nerve block, physical therapy, and the prescribed medication types for each surgery type. In addition, to examine the characteristics of surgeries for patients who underwent two or more surgeries within the same year, further analysis was performed for the following information: type of the first and second surgeries, the interval between the two surgeries, the medical expense of the first and second surgeries, and length of stay (LOS) during hospitalization. 

### 2.4. Statistical Analysis

Descriptive statistical analysis was performed. The proportions of decompression and fusion surgeries out of the total number of claims are represented as percentages, and the yearly trends in the number of claims for each category are presented in a graph to examine the trends in spinal surgeries by year. The total number of claims and expenses for patients who used decompression or fusion surgical services with M480 as the main diagnosis were obtained for each year, and the average expense per claim was calculated by dividing the total expense by the number of claims. In addition, the average medical expenses for decompression or fusion were analyzed for each age group, and the differences were analyzed and presented using graphs. 

The expenses per category and their respective rates out of the total expenses are presented as percentages. The graphs depict yearly trends in the number of claims and cost proportions per category. In cases where patients underwent multiple spinal surgeries within the same year, the proportion of the number of claims is presented as a percentage for each year. The number of claims and medical expenses is also presented as percentages by category, derived from the surgery-related claims. The details of the patient’s treatment for inpatient care for the categories of injection, nerve block, and physical therapy and medications are presented by decompression and fusion, and the proportions of claims by category are presented as percentages. The data are also presented by year, and the proportions are presented as a percentage for each category. The categorical variables were tested by McNemar’s test and the continuous variables were tested by paired *t*-test to compare the characteristics between the first and the second surgeries.

All expenses in Korean won (KRW) were converted to United States dollars (USD) according to the KRW-to-USD exchange rate for each year, and the values were reported after adjusting for the 2019 consumer price index in the healthcare sector. The exchange rate applied each year and the consumer price index in the healthcare service sector in 2019 are summarized in Supplementary Table S1. All statistical analyses were performed using SAS 9.4 (SAS Institute Inc., Cary, NC, USA). A significance level was defined as 0.05 or less.

## 3. Results

### 3.1. Flow Chart

**Figure 1:**
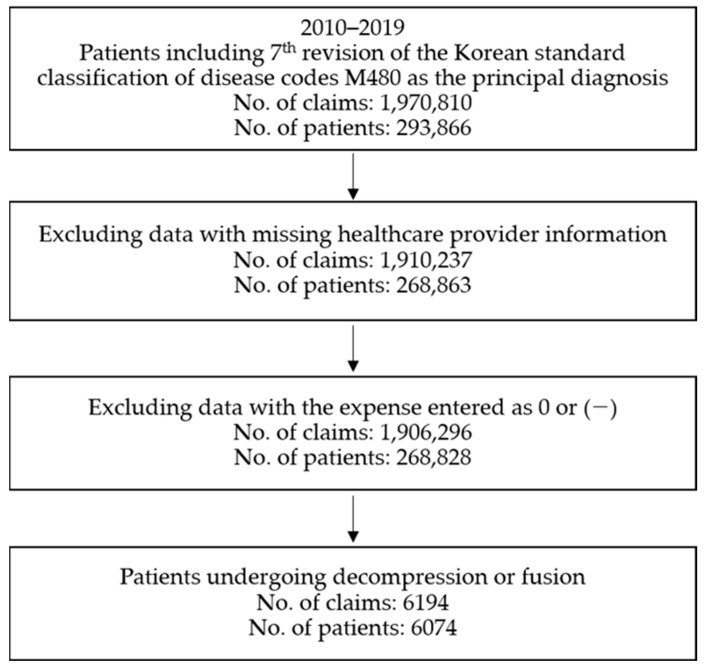
Flow chart of participants.

For 2010–2019, the number of patients who used healthcare services with LSS as the principal diagnosis was 293,866, and the total number of claims was 1,970,810. From these data, those with missing information on healthcare providers or claims with records of expense as 0 or (− were excluded; finally, 6194 claims (6074 patients) showing surgical service utilization of decompression or fusion were used for analysis. (Figure 1).

### 3.2. General Characteristics of the Participants

Table 1 presents the general characteristics of 6194 claims that underwent spinal decompression or fusion surgery with a diagnosis of M480 between 2010 and 2019 in Korea. By age, the age group of 65–74 years accounted for the highest proportions at 37.5% and 43.7% for decompression and fusion, respectively. Regarding sex, both surgery types included more women (54.2% in the decompression group and 59.8% in the fusion group). Most of the spinal surgeries were performed at general hospitals or higher levels of medical institutions; decompression was mainly performed at general hospitals and fusion at tertiary hospitals. 

### 3.3. Overall Trends of Surgical Treatment for LSS

**Figure 2:**
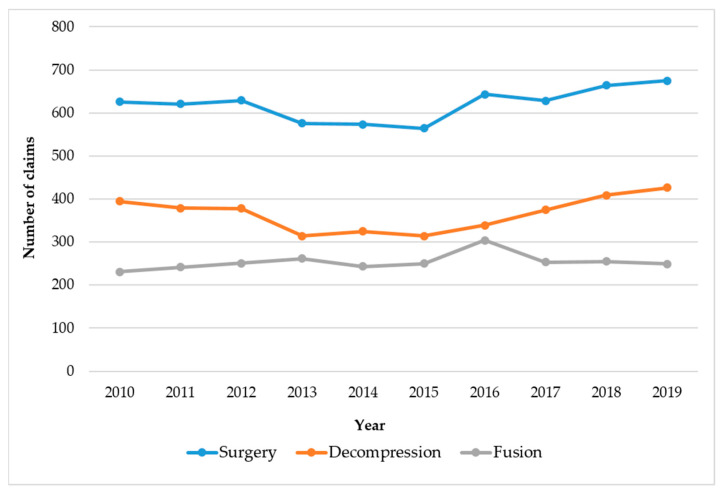
Ten-year trend (2010–2019) in the number of claims for surgeries of patients with LSS.

Figure 2 shows the trend of changes in the number of claims for surgical treatment of patients with LSS for the 10 years from 2010 to 2019. The number of claims for decompression gradually decreased until 2015, followed by a gradual increase from 2016 onwards. The number of claims for spinal fusion was the largest in 2016, but the number gradually decreased. Details of the number of claims for decompression and fusion surgeries in each year are presented in Supplementary Table S2. 

**Figure 3:**
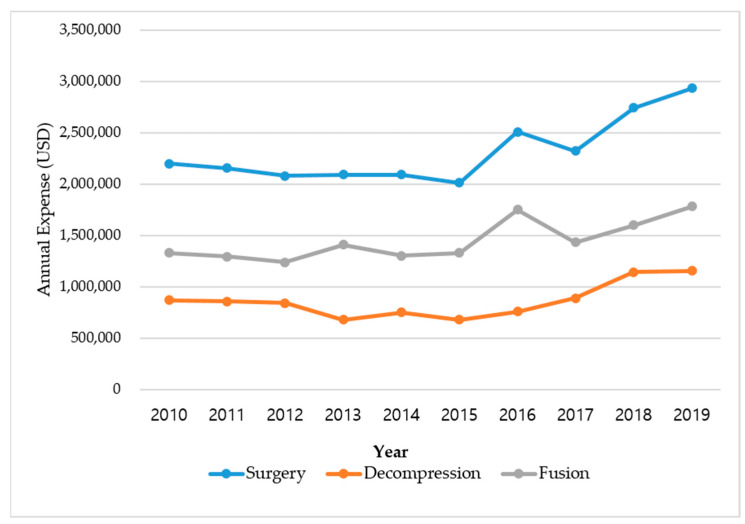
Ten-year trend (2010–2019) in the medical expenses for surgery in patients with LSS.

Figure 3 shows the changes in medical expenses associated with surgery in patients with LSS between 2010 and 2019. In the case of spinal decompression, a decrease from the previous year was observed in 2012, 2014, and 2017; however, for other years, a gradually increasing trend was confirmed. In the case of fusion, a gradual increase in medical expenses has been observed since 2015. Details of the medical expenses for decompression and fusion surgeries in each year are presented in Supplementary Table S2.

### 3.4. Patients Undergoing Two or More Surgeries

Table 2 presents the characteristics of patients who underwent spinal decompression or fusion surgery twice or more in one year. Seventy-nine patients underwent two or more surgeries in one year, and one patient underwent three spinal surgeries in the same year. Most of these patients were female (55.7%), and patients aged 65–74 years accounted for the highest proportion (41.8%).

Table 3 presents the surgical characteristics of 79 patients who underwent two or more surgeries in the same year. For these re-operation cases, decompression accounted for the highest proportion of the first surgery, and the decompression rate showed a significant decrease in the second surgery (*p* = 0.0093). The mean interval between the two surgeries was 88.61 ± 70.16 days. The mean medical expense for the first surgery was USD 3887.92 ± 3171.12, and that of the second surgery was USD 4034.26 ± 3450.92. The mean LOS of the first surgery was 16.27 ± 11.81 days, and that of the second surgery was 17.86 ± 12.57 days. Thus, the medical expenses were higher, and the LOS was longer for the second surgery, but the difference between the two surgeries was not statistically significant. 

### 3.5. Analysis of Medical Service Utilization for the Inpatient Care of LSS Patients Undergoing Spinal Surgery

#### 3.5.1. LOS and Medical Expenses 

Table 4 shows the LOS and expenses of surgical patients according to age and sex. The mean LOS for decompression surgery was 11.55 ± 7.13 days for the age group ≤ 44 years and 14.19 ± 9.16 days for the age group ≤ 75 years, showing an increasing trend in the mean LOS with age. The mean LOS for fusion surgery was 18.92 ± 11.81 days for the age group ≤ 44 years and 19.01 ± 11.61 days for the age group ≥ 75 years, indicating the same trend as that for age, as in the case of decompression surgery. Overall, the LOS for spinal fusion was longer than that for decompression. 

The mean expense for inpatient care also showed an increasing trend with age for decompression (USD 1879.04 ± 7.13 for the age group ≤ 44 years and USD 2732.02 ± 9.16 for the age group ≥ 75 years) and fusion (USD 4283.59 ± 11.81 for the age group ≤ 44 years and USD 6225.33 ± 11.61 for the age group ≥ 75 years). Additionally, the medical expense of fusion surgery was higher than that of decompression surgery, and the difference in the mean expense between fusion and decompression showed an increasing trend with age (Supplementary Figure S1). 

The number of claims and medical expenses for each category of surgery claims are outlined in Supplementary Table S3. Approximately 99% of the total claims showed expenses for radiology (imaging and treatment), and anesthetics accounted for the highest proportion of medical expenses (39.8%).

#### 3.5.2. Details of Treatment Received for Inpatient Care

Table 5 details the treatments received during the hospital stay for spinal surgery. Injections, nerve blocks, and physical therapy were performed in 99.9%, 16.1%, and 44.7% of total claims for surgery, respectively. Among types of injections, intravenous injection accounted for the highest proportion (99.9%) of nerve blocks and epidural nerve blocks (10.5%); among physical therapies, electric therapy (36.8%), and heat/cold therapy (36.5%) accounted for the highest proportion, respectively. Regarding trends according to the type of surgery, the prescription rates for heat/cold and electrical therapies were higher for decompression surgery than for spinal fusion.

Supplementary Table S4 shows the annual prescription rates for injections, nerve blocks, and physical therapy. Among the prescription rates, a noteworthy trend was a rapid increase in the prescription rate of nerve block from 7.2% in 2011 to 19.1% in 2012. In addition, the prescription rate of nerve blocks was approximately 20% on average after that but decreased to 12.4% in 2017. 

#### 3.5.3. Details of Prescribed Medications during Inpatient Care

Table 6 shows the proportions of medications prescribed for inpatient care, including opioids, non-opioid analgesics, anesthetics, gastrointestinal medications, antipsychotics, antibiotics, and steroids. Opioids were used in 88.5% of decompression and 97.7% of fusion cases, whereas non-opioid analgesics were used in 93.6% of decompression and 98.1% of fusion cases. There was no difference in the prescription rates for most medications by surgery type, but opioids (97.7% vs. 88.5%) and antibiotics (79.4% vs. 66.8%) were prescribed more frequently for fusion surgery than decompression surgery. 

**Figure 4:**
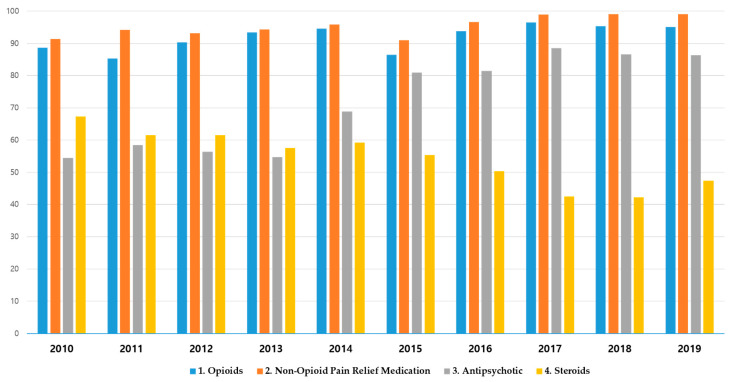
Yearly trends in the prescription rates of medications.

Figure 4 shows the percentage of prescriptions per year for each type of medication. The prescription rate of opioids decreased in 2015 but increased thereafter. The prescription rate of steroids decreased over time, whereas that of antipsychotics increased. The consumption of different medications each year is presented in Supplementary Table S5.

## 4. Discussion

This study analyzed the trends of medical service utilization in patients diagnosed with LSS (M480) undergoing surgical fusion or decompression using the claims data of the HIRA-NPS from 2010 to 2019. 

Decompression procedures exhibited a consistent upward trend throughout the study period, while fusion procedures displayed a decreasing trend from 2016. Despite the risk of fusion that has been reported in the past [18], there was a tendency to prefer fusion, a highly complicated surgical technique, in cases with spinal instability [13,19,20]. Consequently, fusion has risen steeply compared to decompression [21]. However, there have been continuous studies on the risks of fusion, which show no significant clinical benefits of this technique [22,23]. Therefore, the recent trend of decreasing fusion rates in the results of this study can be interpreted as reflecting those conducted recently.

Decompression was performed more frequently in general than in tertiary hospitals, whereas fusion was performed more frequently in tertiary than in general hospitals. The findings indicate that patients tend to visit general hospitals for simpler surgeries but prefer tertiary hospitals for surgeries with greater complexity. In addition, increased accessibility to tertiary hospital is thought to be one of the reasons for the higher rate of fusion in tertiary hospitals.

In this study, the LOS and medical expenses of inpatient care related to spinal surgery were higher in women than in men and increased with age. LOS in the post-anesthesia care unit (PACU) and medical expenses can be examined in conjunction with the patient’s condition during their stay. A longer duration of instability in the patient’s condition can result in an extended LOS in the PACU, which may contribute to a slower recovery process and increased medical expenses [24]. According to a previous study, the length of stay in the PACU was longer in women than in men and increased with age [24]. In addition, fusion involves more surgical complexity than decompression and causes damage to various anatomical structures [25]; therefore, a longer recovery time is required for fusion regardless of the age and sex of the patients, resulting in longer LOS and greater expense of inpatient care. Furthermore, the additional medical service remuneration rates based on the type of medical institution of the national health insurance system of Korea are 30% for tertiary hospitals and 25% for general hospitals, indicating a higher expense of care in tertiary hospitals than in general hospitals. It may be associated with higher expense for fusion, which is performed more frequently in tertiary hospitals than in general hospitals. 

This study analyzed the data of patients who underwent two or more surgeries in the same year as a surrogate measure of re-operation. In the first surgery, decompression was 31.6% more common than fusion, but in the second surgery, fusion was 1.2% more common than decompression. This trend suggests a preference for a more complex surgical approach during subsequent surgeries. A previous study related to re-operation showed that fusion had a higher four-year re-operation rate (1.7%) than decompression [26]. In another study, the five-year re-operation rate for LSS surgery was 14.2% and showed no significant difference between decompression and fusion [27,28]. If this rate is compared to the range of re-operation rates reported in previous studies (11~17%) [29,30], it can be reasoned that the overall re-operation rate increased.

For medications used, opioids and non-opioid analgesics each had a prescription rate of more than 90%, and antipsychotics also showed a prescription rate of more than 80% since 2015. This prescription rate refers to the ratio of claims for which the drug was prescribed out of all claims of surgery. Therefore, the high prescription rate of each drug means that a patient received multiple types of drug prescriptions during hospitalization. In particular, it is expected that most of the patients were prescribed opioids, non-opioid analgesics, and antipsychotics. 

Although opioid therapy is highly effective in pain management, the risk of side effects is substantial, highlighting the necessity for serious consideration of potential harm [31]. In a previous study, patients aged >65 years with chronic musculoskeletal pain who used opioids reported that the risk of adverse events was three times higher than that of the control group, but opioids only had a small effect on decreasing pain intensity [32]. Another study reported that more than 33,000 people died from opioid overdoses across the U.S. in 2015 [33], and another study reported that the prolonged use of opioids increased the prevalence of opioid dependence in 20% of spine surgery patients [34]. 

Despite those risks, in the U.S., the number of patients prescribed opioids for lower back pain has doubled over the past 10 years [35]. In addition, 77% of patients who underwent surgical treatment for hip fractures [36] and 82% of patients who underwent surgery for ankle fractures in the U.S. were prescribed opioids after surgery [36,37]. In this study, more than 90% of the patients were prescribed opioids, indicating a potentially high risk of using opioids.

Antipsychotics are a type of medication known to be effective for managing chronic and neuropathic pain. They are used as adjuvant analgesics for pain management in patients with dependence, tolerance, or side effects to opioids [38]. The results of our study showed that the prescription of antipsychotics increased sharply in 2014 compared to previous years and continued to show a steady increase after that. However, antipsychotic prescriptions require caution because of the potential side effects of drowsiness and reduced concentration, resulting in falls or cardiovascular diseases [39]. Steroids are also an adjuvant analgesic mainly used as an anti-inflammatory medicine; however, side effects such as osteoporosis, osteonecrosis, myopathy, gastrointestinal disorders, and adverse influences on the cardiovascular system have been reported [40]. Thus, the medication should be used carefully to maximize the effect of reducing inflammation while minimizing possible side effects. 

### Strengths and Limitations

Attention should be paid to the interpretation of the results of this study for several reasons. First, this study analyzed sample data rather than the entire population of Korea. Although it is judged that it can represent the entire population because a statistical sampling process was used, care must be taken when interpreting the total number of patients, the number of claims, and expenditure. 

Next, since this study used secondary data; the analysis was limited to the details of healthcare service utilization that could be known from this data. The absence of evaluating clinical symptoms and severity in patients with LSS was also a major drawback of this study. Thus, in future research, an analysis of the trends in surgical service utilization according to clinical symptoms and severity of LSS is needed. In addition, the treatments not covered by NHI could not be analyzed. Including the treatment only covered by the NHI may have led to an underestimation of the actual medical expenses involved. 

Finally, the data used in this study were cross-sectional data segmented by year, and, although the overall status and trends could be investigated, the individual changes or long-term follow-up could not be analyzed. Therefore, we could not analyze re-operation, but we analyzed patients who underwent two or more surgeries. 

Nevertheless, this study showed a recent 10-year trend in Korean surgical service utilization for patients diagnosed with LSS using HIRA-NPS data between 2010 and 2019. Furthermore, hospitalization costs, detailed treatment administered, and medication prescribed during hospitalization are newly provided. The findings of this study are expected to provide basic research data for clinicians, researchers, and policymakers. 

## 5. Conclusions

Analyzing sample data from Korea, both decompression and fusion surgeries in LSS patients increased in 2019 compared to 2010, but from the mid-2010s, fusion surgeries have gradually decreased and decompression surgeries increased. The prescribing rate for opioids and non-opioid analgesics was more than 90%, and this also slightly increased. Future studies should address the limitations associated with claims data and the sampled data utilized in the present study.

## Figures and Tables

**Table 1:** Basic characteristics of the claims.

	Total	Type of Surgery		
		Decompression	Fusion	*p*-Value
**Total**	6194 (100)	3654 (100)	2540 (100)	
**Age (years)**	**□**	**□**	**□**	<0.0001
≤44	203 (3.3)	165 (4.5)	38 (1.5)	
45–54	675 (10.9)	432 (11.8)	243 (9.6)	
55–64	1713 (27.7)	990 (27.1)	723 (28.5)	
65–74	2482 (40.1)	1371 (37.5)	1111 (43.7)	
≥75	1121 (18.1)	696 (19.0)	425 (16.7)	
**Sex**	**□**	**□**	**□**	<0.0001
Male	2696 (43.5)	1675 (45.8)	1021 (40.2)	
Female	3498 (56.5)	1979 (54.2)	1519 (59.8)	
**Payer type**	**□**	**□**	**□**	<0.0001
National Health Insurance	5680 (91.7)	3406 (93.2)	2274 (89.5)	
Medicaid	479 (7.7)	228 (6.2)	251 (9.9)	
Others	35 (0.6)	20 (0.5)	15 (0.6)	
**Type of visit**	**□**	**□**	**□**	0.0007
Outpatient	15 (0.2)	15 (0.4)	0 (0.0)	
Inpatient	6179 (99.8)	3639 (99.6)	2540 (100)	
**Medical institutions**	**□**	**□**	**□**	<0.0001
Tertiary hospitals	2649 (42.8)	1033 (28.3)	1616 (63.6)	
General hospitals	3449 (55.7)	2545 (69.6)	904 (35.6)	
Hospitals or local clinics	96 (1.5)	76 (2.1)	20 (0.8)	

Differences were tested using chi-square test or Fisher’s exact test.

**Table 2:** Characteristics of patients who underwent more than two surgeries in the same year.

Category	No. of Patients, *n* (%)
Total number of patients	79
Number of surgeries	□
Twice	78 (98.7)
Thrice	1 (1.3)
Year	□
2010	4 (5.1)
2011	3 (3.8)
2012	8 (10.1)
2013	9 (11.4)
2014	9 (11.4)
2015	9 (11.4)
2016	7 (8.9)
2017	5 (6.3)
2018	13 (16.5)
2019	12 (15.2)
Sex	□
Male	35 (44.3)
Female	44 (55.7)
Age (Years)	□
≤44	1 (1.3)
45–54	11 (13.9)
55–64	23 (29.1)
65–74	33 (41.8)
≥75	11 (13.9)

**Table 3:** Characteristics of surgeries for patients undergoing two or more surgeries in the same year.

Characteristics of Surgery	Values	*p*-Value
**Type of the first surgery, N (%)**	**□**	0.0093 ^1^
Decompression	52 (65.8)	
Fusion	27 (34.2)	
**Type of the second surgery, N (%)**	**□**	
Decompression	39 (49.4)	
Fusion	40 (50.6)	
**Interval between the two surgeries (days)**	**□**	
Mean ± SD	88.61 ± 70.16	
Median(IQR)	67 (35, 126)	
**Medical expense (USD)**	**□**	0.7404 ^2^
First surgery	3887.92 ± 3171.12	
Second surgery	4034.26 ± 3450.92	
**Length of stay (days)**	**□**	0.3406 ^2^
First surgery	16.27 ± 11.81	
Second surgery	17.86 ± 12.57	

^1^ McNemar’s test was used to calculate the *p*-value. ^2^ The paired *t*-test was used to calculate the *p*-value. IQR, interquartile range; SD, standard deviation; USD, US dollars.

**Table 4:** Length of stay and medical expenses for the two LSS surgeries by patients’ demographic characteristics.

Category	Total	Type of Surgery	
		Decompression	Fusion
**Length of Stay (Days)**			
**Age**	**□**	**□**	**□**
≤44	12.93 ± 8.67	11.55 ± 7.13	18.92 ± 11.81
45–54	14.24 ± 8.22	12.53 ± 7.37	17.27 ± 8.78
55–64	14.41 ± 8.48	12.58 ± 7.75	16.91 ± 8.80
65–74	15.56 ± 9.37	13.15 ± 7.81	18.53 ± 10.24
≥75	16.02 ± 10.42	14.19 ± 9.16	19.01 ± 11.61
**Sex**	**□**	**□**	**□**
Male	13.94 ± 8.77	11.97 ± 7.36	17.17 ± 9.89
Female	15.98 ± 9.46	13.96 ± 8.43	18.61 ± 10.07
**Medical expense (USD)**			
**Age**			
≤44	2329.15 ± 8.67	1879.04 ± 7.13	4283.59 ± 11.81
45–54	2988.59 ± 8.22	2039.98 ± 7.37	4675.02 ± 8.78
55–64	3513.05 ± 8.48	2164.21 ± 7.75	5360.00 ± 8.80
65–74	4041.33 ± 9.37	2468.05 ± 7.81	5982.78 ± 10.24
≥75	4056.42 ± 10.42	2732.02 ± 9.16	6225.33 ± 11.61
Sex	**□**	**□**	**□**
Male	3434.28 ± 8.77	2239.85 ± 7.36	5393.79 ± 9.89
Female	3952.82 ± 9.46	2459.48 ± 8.43	5898.39 ± 10.07

**Table 5:** Prescription rates of injection, nerve block, and physical therapy by surgery type.

	Total	Type of Surgery	
		Decompression	Fusion
**□**	**N (%)**	**N (%)**	**N (%)**
**Injection**	**□**	**□**	**□**
Subcutaneous or intramuscular injection	5426 (87.6)	3179 (87.0)	2247 (88.5)
Intravenous injection	6186 (99.9)	3646 (99.8)	2540 (100.0)
Intra articular injection	146 (2.4)	90 (2.5)	56 (2.2)
Total	6187 (99.9)	3647 (99.8)	2540 (100.0)
**Nerve block**	**□**	**□**	**□**
Epidural block	650 (10.5)	376 (10.3)	274 (10.8)
Peripheral branch block	70 (1.1)	40 (1.1)	30 (1.2)
Spinal nerve plexus, root or ganglion block	381 (6.2)	245 (6.7)	136 (5.4)
Sympathetic plexus or ganglion block	2 (0.0)	- (0.0)	2 (0.1)
Total	996 (16.1)	592 (16.2)	404 (15.9)
**Physical therapy**	**□**	**□**	**□**
Heat/cold therapy	2262 (36.5)	1526 (41.8)	736 (29.0)
Electric therapy	2277 (36.8)	1528 (41.8)	749 (29.5)
Trigger point injection therapy	159 (2.6)	89 (2.4)	70 (2.8)
Exercise therapy	663 (10.7)	347 (9.5)	316 (12.4)
Traction therapy	48 (0.8)	35 (1.0)	13 (0.5)
Laser therapy	374 (6.0)	263 (7.2)	111 (4.4)
Total	2771 (44.7)	1771 (48.5)	1000 (39.4)
**Total**	6194 (100.0)	3654 (100.0)	2540 (100.0)

**Table 6:** Prescription rate of medications by surgery type.

□	Total	Type of Surgery	
□		Decompression	Fusion
	**N (%)**	**N (%)**	**N (%)**
**Opioids**	**5717 (92.3)**	**3235 (88.5)**	**2482 (97.7)**
**Non-opioid analgesics**	**□**	**□**	**□**
Non-steroidal anti-inflammatory drugs	5077 (82.0)	2898 (79.3)	2179 (85.8)
Neuralgia medication	2332 (37.6)	1065 (29.1)	1267 (49.9)
Muscle relaxants	4182 (67.5)	2349 (64.3)	1833 (72.2)
Others	2148 (34.7)	1091 (29.9)	1057 (41.6)
Total	5913 (95.5)	3421 (93.6)	2492 (98.1)
**Anesthetics**	5679 (91.7)	3342 (91.5)	2337 (92.0)
**Gastrointestinal medications**	6110 (98.6)	3584 (98.1)	2526 (99.4)
**Antipsychotics**	4460 (72.0)	2442 (66.8)	2018 (79.4)
**Antibiotics**	**□**	**□**	**□**
Systemic antibiotics	255 (4.1)	104 (2.8)	151 (5.9)
Topical antibiotics	5331 (86.1)	2971 (81.3)	2360 (92.9)
Total	4460 (72.0)	2442 (66.8)	2018 (79.4)
**Steroids**	**□**	**□**	**□**
Systemic steroids	293 (4.7)	131 (3.6)	162 (6.4)
Topical steroids	3229 (52.1)	1726 (47.2)	1503 (59.2)
Total	3360 (54.2)	1796 (49.2)	1564 (61.6)
**Total**	6194 (100.0)	3654 (100.0)	2540 (100.0)

## Data Availability

The datasets generated and analyzed in the current study are available in the HIRA-NPS repository. The study utilized HIRA data, which are third-party data and thus not owned by the authors. The HIRA data are available upon direct request, via email or fax, and submission of the request form and declaration of data use, which are downloadable from the HIRA website [http://opendata.hira.or.kr] upon payment of a data request fee (KRW 300,000 per dataset).

## Supplementary Materials

The following supporting information can be downloaded at: https://www.mdpi.com/article/10.3390/medicina59091582/s1, Figure S1: Medical expenses of patients with lumbar spinal stenosis undergoing surgery by age group; Table S1: KRW to USD exchange rate and the consumer price index for each year; Table S2: General medical service use by patients with spinal stenosis in Korea; Table S3: Summary of the number of spinal surgery claims and medical expenses by category; Table S4: Prescription rates of injections, nerve blocks, and physical therapy by year; Table S5: Prescription rates for different types of medications by year.
