# Supplementary material for: Trends of Surgical Service Utilization for Lumbar Spinal Stenosis in South Korea: A 10-Year (2010–2019) Cross-Sectional Analysis of the Health Insurance Review and Assessment Service—National Patient Sample Data
Source: Medicina (Kaunas). 2023 Aug 31;59(9):1582. doi: 10.3390/medicina59091582 (PMC10533068; doi:10.3390/medicina59091582)
Supplement: Supplementary file 1 [file medicina-59-01582-s001.zip › JASME_198_Supplementary_figure.pdf]

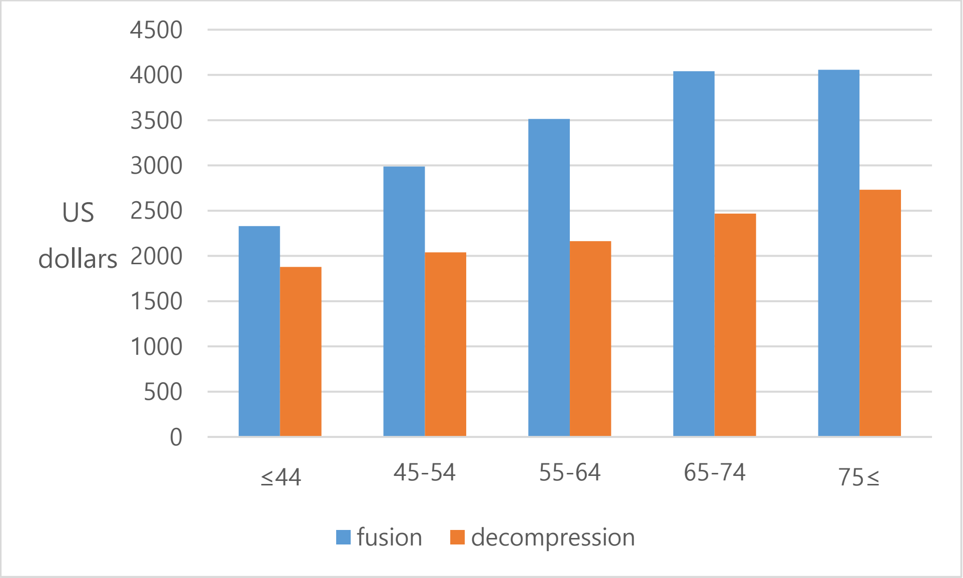

(A)

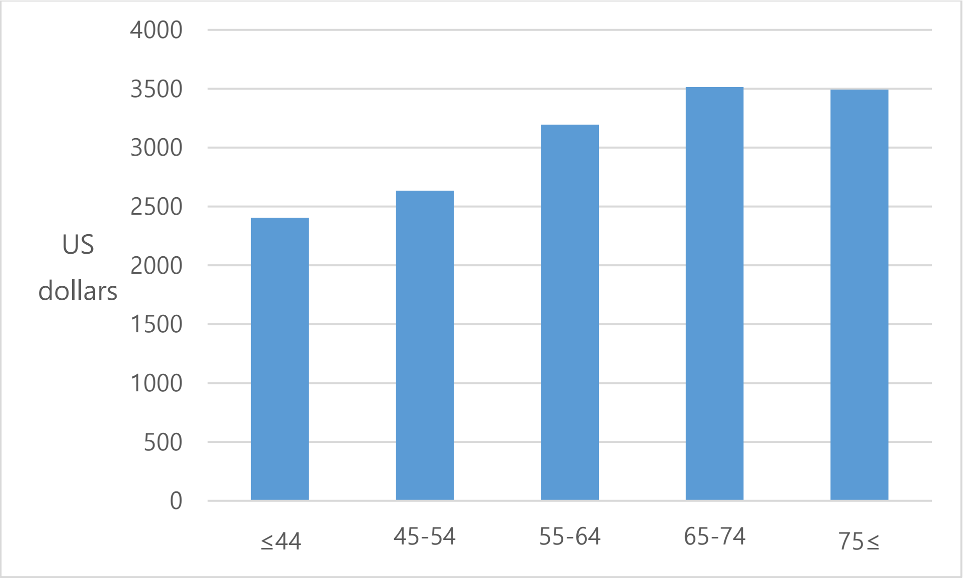

(B)

Figure S1. Medical expenses of patients with lumbar spinal stenosis undergoing surgery by age group. (A) Medical expenses for spinal decompression and fusion surgery by age group. (B) Difference in medical expenses between spinal fusion and decompression surgery by age group.
