# Supplementary material for: Trends of Surgical Service Utilization for Lumbar Spinal Stenosis in South Korea: A 10-Year (2010–2019) Cross-Sectional Analysis of the Health Insurance Review and Assessment Service—National Patient Sample Data
Source: Medicina (Kaunas). 2023 Aug 31;59(9):1582. doi: 10.3390/medicina59091582 (PMC10533068; doi:10.3390/medicina59091582)
Supplement: Supplementary file 1 [file medicina-59-01582-s001.zip › JASME_198_Supplementary_tables.pdf]

Table S1. KRW to USD exchange rate and the consumer price index for each year.

Year	Exchange rate	Price
2010	1,156	0.930244
2011	1,107.99	0.946598
2012	1,126.76	0.955279
2013	1,095.04	0.95869
2014	1,053.12	0.965445
2015	1,131.52	0.977517
2016	1,160.41	0.987195
2017	1,130.48	0.995894
2018	1,100.58	0.995406
**2019**	**1,166.11**	**1**

Table S2. General medical service use by patients with spinal stenosis in Korea

YEAR		Total patients	Total claims	Annual expense†[1]	Avg. expense per claim†
2010	Subtotal	19,010	112,575	6,205,748.95	**55.13**
	surgery	622	626	2,197,989.68	**3,511.17**
	decompression	393	395	867,549.31	**2,196.33**
	fusion	230	231	1,330,440.37	**5,759.48**
2011	Subtotal	21,933	130,261	6,790,538.28	**52.13**
	surgery	613	621	2,153,047.19	**3,467.06**
	decompression	376	379	857,910.71	**2,263.62**
	fusion	239	242	1,295,136.48	**5,351.8**
2012	Subtotal	24,920	166,226	7,503,591.19	**45.14**
	surgery	621	629	2,078,636.32	**3,304.67**
	decompression	376	378	840,126.09	**2,222.56**
	fusion	247	251	1,238,510.23	**4,934.3**
2013	Subtotal	26,725	179,544	8,344,032.49	**46.47**
	surgery	562	576	2,090,822.38	**3,629.9**
	decompression	309	314	680,368.63	**2,166.78**
	fusion	255	262	1,410,453.75	**5,383.41**
2014	Subtotal	28,674	196,066	9,297,114.38	**47.42**
	surgery	562	573	2,091,511.11	**3,650.11**
	decompression	321	325	751,712.08	**2,312.96**
	fusion	238	243	1,302,324.67	**5,359.36**
2015	Subtotal	30,238	206,250	9,513,847.5	**46.13**
	surgery	552	564	2,009,866.31	**3,563.59**
	decompression	307	314	679,489.94	**2,163.98**
	fusion	249	250	1,330,376.37	**5,321.51**
2016	Subtotal	32,388	225,440	10,667,098.84	**47.32**
	surgery	632	643	2,507,041.06	**3,898.98**
	decompression	336	339	756,615.07	**2,231.9**
	fusion	300	304	1,750,425.99	**5,757.98**
2017	Subtotal	34,418	233,073	11,254,829.37	**48.29**
	surgery	622	628	2,321,011.16	**3,695.88**
	decompression	372	375	890,109.67	**2,373.63**
	fusion	252	253	1,430,901.49	**5,655.74**
2018	Subtotal	36,705	248,193	12,778,671.78	**51.49**

	surgery	649	664	2,740,227.29	**4,126.85**
	decompression	401	409	1,142,103.72	**2,792.43**
	fusion	252	255	1,598,123.57	**6,267.15**
2019	Subtotal	38,793	268,926	13,924,605.79	**51.78**
	surgery	659	675	2,933,105.42	**4,345.34**
	decompression	417	426	1,153,078.94	**2,706.76**
	fusion	244	249	1,780,026.48	**7,148.7**
Total	Subtotal	1,906,296	268,828	96,280,078.57	**48.9588**
	surgery	6,074	6,194	23,085,783.56	**3,727.12**
	decompression	4,915	4,993	16,301,166.3	**3,264.8**
	fusion	1,195	1,201	6,784,617.26	**5,649.14**

†All expenditures were converted based on the annual average exchange rate (KRW/USD), and the price is adjusted to the health expenditure price level of 2019. (see Supplementary Table 1 for further detail).

Table S3. Summary of the number of spinal surgery claims and medical expenses by category

No. of claims, N (%)	Total		Type of surgery			
			Decompression		Fusion	
**Examination**	5,535 (89.4)	1,710,459 (6.8)	3,255 (89.1)	741,191 (7.9)	2,280 (89.8)	969,268 (6.1)
**Injection fee**	5,557 (89.7)	1,963 (0.0)	3,275 (89.6)	513 (0.0)	2,282 (89.8)	1,450 (0.0)
**Medication**	5,532 (89.3)	546,477 (2.2)	3,257 (89.1)	216,180 (2.3)	2,275 (89.6)	330,297 (2.1)
**Treatment**	5,561 (89.8)	2,063,465 (8.2)	3,279 (89.7)	860,491 (9.1)	2,282 (89.8)	1,202,974 (7.6)
**Admission fee**	5,548 (89.6)	506,712 (2.0)	3,262 (89.3)	253,202 (2.7)	2,286 (90.0)	253,509 (1.6)
**Anesthetics**	5,534 (89.3)	10,066,321 (39.8)	3,259 (89.2)	2,636,171 (28.0)	2,275 (89.6)	7,430,150 (46.8)
**Radiology**	6,125 (98.9)	4,672,805 (18.5)	3,607 (98.7)	2,266,632 (24.0)	2,518 (99.1)	2,406,173 (15.2)
**Diagnostic**	5,526 (89.2)	1,713,329 (6.8)	3,259 (89.2)	779,050 (8.3)	2,267 (89.3)	934,280 (5.9)
**Physical therapy**	2,498 (40.3)	904,409 (3.6)	1,596 (43.7)	461,753 (4.9)	902 (35.5)	442,655 (2.8)
**Psychological therapy**	84 (1.4)	328,985 (1.3)	21 (0.6)	136,340 (1.4)	63 (2.5)	192,645 (1.2)
**Non benefit and others**	2,080 (33.6)	130,005 (0.5)	1,221 (33.4)	73,540 (0.8)	859 (33.8)	56,466 (0.4)
**Total**	6,194 (100.0)	25,309,981 (100.0)	3,654 (100.0)	9,428,796 (100.0)	2,540 (100.0)	15,881,185 (100.0)

Table S4. Prescription rates of injections, nerve blocks, and physical therapy by year

	Total	2010	2011	2012	2013	2014	2015	2016	2017	2018	2019
**Injection**	**6,187 (99.9)**	**626 (100.0)**	**621 (100.0)**	**629 (100.0)**	**575 (99.8)**	**567 (99.0)**	**560 (99.3)**	**643 (100.0)**	**628 (100.0)**	**664 (100.0)**	**674 (99.9)**
subcutaneous or intramuscular injection	5,426 (87.6)	594 (94.9)	582 (93.7)	565 (89.8)	503 (87.3)	493 (86.0)	495 (87.8)	557 (86.6)	532 (84.7)	547 (82.4)	558 (82.7)
intravenous injection	6,186 (99.9)	626 (100.0)	621 (100.0)	629 (100.0)	575 (99.8)	567 (99.0)	560 (99.3)	643 (100.0)	628 (100.0)	664 (100.0)	673 (99.7)
Intra articular injection	146 (2.4)	17 (2.7)	14 (2.3)	15 (2.4)	15 (2.6)	13 (2.3)	9 (1.6)	14 (2.2)	15 (2.4)	20 (3.0)	14 (2.1)
**Nerve block**	**996 (16.1)**	**68 (10.9)**	**45 (7.2)**	**120 (19.1)**	**131 (22.7)**	**143 (25.0)**	**110 (19.5)**	**145 (22.6)**	**78 (12.4)**	**78 (11.7)**	**78 (11.6)**
Epidural block	650 (10.5)	36 (5.8)	18 (2.9)	86 (13.7)	105 (18.2)	111 (19.4)	87 (15.4)	109 (17.0)	42 (6.7)	28 (4.2)	28 (4.1)
Peripheral branch block	70 (1.1)	5 (0.8)	6 (1.0)	7 (1.1)	5 (0.9)	9 (1.6)	6 (1.1)	7 (1.1)	6 (1.0)	8 (1.2)	11 (1.6)
Spinal nerve plexus, root, or ganglion block	381 (6.2)	35 (5.6)	25 (4.0)	37 (5.9)	36 (6.3)	34 (5.9)	28 (5.0)	41 (6.4)	45 (7.2)	54 (8.1)	46 (6.8)
Sympathetic plexus or ganglion block	2 (0.0)	0 (0.0)	0 (0.0)	0 (0.0)	1(0.2)	0 (0.0)	1(0.2)	0 (0.0)	0 (0.0)	0 (0.0)	0 (0.0)
**Physical therapy**	**2,771 (44.7)**	**283 (45.2)**	**265 (42.7)**	**277 (44.0)**	**243 (42.2)**	**242 (42.2)**	**243 (43.1)**	**299 (46.5)**	**282 (44.9)**	**319 (48.0)**	**318 (47.1)**
heat/cold therapy	2,262 (36.5)	222 (35.5)	218 (35.1)	226 (35.9)	192 (33.3)	196 (34.2)	199 (35.3)	244 (37.9)	233 (37.1)	268 (40.4)	264 (39.1)
electric therapy	2,277 (36.8)	229 (36.6)	212 (34.1)	225 (35.8)	189 (32.8)	196 (34.2)	199 (35.3)	247 (38.4)	233 (37.1)	273 (41.1)	274 (40.6)
Trigger point injection therapy	159 (2.6)	14 (2.2)	12 (1.9)	28 (4.5)	11 (1.9)	16 (2.8)	18 (3.2)	14 (2.2)	15 (2.4)	16 (2.4)	15 (2.2)
exercise therapy	663 (10.7)	84 (13.4)	63 (10.1)	59 (9.4)	67 (11.6)	59 (10.3)	52 (9.2)	87 (13.5)	56 (8.9)	73 (11.0)	63 (9.3)
traction therapy	48 (0.8)	4 (0.6)	5 (0.8)	3 (0.5)	3 (0.5)	4 (0.7)	5 (0.9)	4 (0.6)	7 (1.1)	7 (1.1)	6 (0.9)
laser therapy	374 (6.0)	42 (6.7)	30 (4.8)	25 (4.0)	36 (6.3)	22 (3.8)	34 (6.0)	50 (7.8)	44 (7.0)	50 (7.5)	41 (6.1)
**Total**	**6,194 (100.0)**	**626 (100.0)**	**621 (100.0)**	**629 (100.0)**	**576 (100.0)**	**573 (100.0)**	**564 (100.0)**	**643 (100.0)**	**628 (100.0)**	**664 (100.0)**	**675 (100.0)**

Table S5. Prescription rates for different types of medications by year

	Total	2010	2011	2012	2013	2014	2015	2016	2017	2018	2019
**Opioids**	5,722 (92.4)	555 (88.7)	530 (85.3)	568 (90.3)	538 (93.4)	542 (94.6)	505 (89.5)	603 (93.8)	606 (96.5)	633 (95.3)	642 (95.1)
**Non-Opioid Pain Relief Medication**	5,918 (95.5)	572 (91.4)	585 (94.2)	586 (93.2)	543 (94.3)	549 (95.8)	513 (91.0)	621 (96.6)	622 (99.0)	658 (99.1)	669 (99.1)
NSAIDS	5,082 (82.0)	473 (75.6)	488 (78.6)	500 (79.5)	461 (80.0)	483 (84.3)	458 (81.2)	541 (84.1)	524 (83.4)	560 (84.3)	594 (88.0)
neuralgia medication	2,336 (37.7)	173 (27.6)	200 (32.2)	187 (29.7)	203 (35.2)	187 (32.6)	205 (36.3)	288 (44.8)	302 (48.1)	298 (44.9)	293 (43.4)
muscle relaxants	4,183 (67.5)	354 (56.5)	361 (58.1)	388 (61.7)	368 (63.9)	368 (64.2)	337 (59.8)	444 (69.1)	509 (81.1)	537 (80.9)	517 (76.6)
Others	2,151 (34.7)	128 (20.4)	123 (19.8)	129 (20.5)	113 (19.6)	121 (21.1)	123 (21.8)	315 (49.0)	343 (54.6)	371 (55.9)	385 (57.0)
**Anesthetic**	5,684 (91.8)	599 (95.7)	598 (96.3)	609 (96.8)	556 (96.5)	549 (95.8)	546 (96.8)	578 (89.9)	549 (87.4)	525 (79.1)	575 (85.2)
**Gastrointestinal**	6,115 (98.7)	621 (99.2)	616 (99.2)	629 (100.0)	575 (99.8)	570 (99.5)	561 (99.5)	632 (98.3)	607 (96.7)	650 (97.9)	654 (96.9)
**Antipsychotic**	4,463 (72.1)	341 (54.5)	363 (58.5)	355 (56.4)	315 (54.7)	395 (68.9)	456 (80.9)	524 (81.5)	556 (88.5)	575 (86.6)	583 (86.4)
**Antibiotics**	5,348 (86.3)	539 (86.1)	520 (83.7)	523 (83.1)	486 (84.4)	484 (84.5)	492 (87.2)	552 (85.8)	561 (89.3)	591 (89.0)	600 (88.9)
Systemic antibiotics	255 (4.1)	18 (2.9)	15 (2.4)	19 (3.0)	19 (3.3)	15 (2.6)	19 (3.4)	33 (5.1)	36 (5.7)	33 (5.0)	48 (7.1)
topical antibiotics	5,335 (86.1)	539 (86.1)	519 (83.6)	520 (82.7)	482 (83.7)	483 (84.3)	492 (87.2)	551 (85.7)	560 (89.2)	590 (88.9)	599 (88.7)
**Steroids**	3,363 (54.3)	421 (67.3)	382 (61.5)	387 (61.5)	331 (57.5)	339 (59.2)	312 (55.3)	324 (50.4)	267 (42.5)	280 (42.2)	320 (47.4)
Systemic steroids	293 (4.7)	19 (3.0)	22 (3.5)	27 (4.3)	20 (3.5)	16 (2.8)	24 (4.3)	41 (6.4)	35 (5.6)	45 (6.8)	44 (6.5)
Topical steroids	3,232 (52.2)	419 (66.9)	371 (59.7)	378 (60.1)	323 (56.1)	337 (58.8)	301 (53.4)	305 (47.4)	245 (39.0)	256 (38.6)	297 (44.0)
**Total**	6,194 (100.0)	626 (100.0)	621 (100.0)	629 (100.0)	576 (100.0)	573 (100.0)	564 (100.0)	643 (100.0)	628 (100.0)	664 (100.0)	675 (100.0)
